# Scaling up! Staff e-learning for a national take-home naloxone program

**DOI:** 10.3389/fdgth.2024.1404646

**Published:** 2024-09-17

**Authors:** Øystein Bruun Ericson, Desiree Eide, Håvar Brendryen, Philipp Lobmaier, Thomas Clausen

**Affiliations:** ^1^ Norwegian Centre for Addiction Research, Institute of Clinical Medicine, University of Oslo, Ullevål Hospital, Oslo, Norway; ^2^ Department of Psychology, Faculty of Social Sciences, University of Oslo, Oslo, Norway; ^3^ Division of Mental Health and Substance Abuse, Diakonhjemmet Hospital, Oslo, Norway

**Keywords:** staff training, overdose prevention, e-health, public health, scaling up

## Abstract

**Background:**

A staff e-learning course was developed to prepare for scaling up a national take-home naloxone (THN) program in Norway. The aims of the study were to (a) describe participant characteristics for those that completed a THN e-learning course, (b) compare opioid overdose knowledge scores before and after e-learning course completion, and (c) to explore subsequent THN distribution by those trained.

**Methods:**

This was a quasi-experimental pre-test, post-test longitudinal cohort study of individuals completing a THN e-learning course from April 2021 to May 2022. Frequency analyses were performed for participant characteristics and subsequent naloxone distributions at 1-week and 1-month follow-up. The opioid overdose knowledge scale (OOKS) was used to measure pre-test-post-test knowledge among participants. Wilcoxon signed-rank test was performed for comparison between pre-test and post-test. Effect size was calculated using Cohen criteria.

**Results:**

In total, 371 individuals were included in this study. Most were either nurses or social workers (*n* = 277, 75%). Participant knowledge increased by medium or large effect for all items measured. At 1-month follow-up, 15% reported naloxone distribution. During the study period, 94 naloxone kits were distributed. Major reasons for not distributing were “clients not interested”, “workplace not distributing” and “workplace in process of distributing”.

**Conclusions:**

Our findings suggest that an e-learning course is equally effective in terms of knowledge transfer as an in-person classroom setting, and may provide engagement in terms of naloxone distribution. However, our findings also emphasize the importance of clear implementation routines, including support from central coordinators to optimize the implementation process.

## 1 Introduction

Over the last 30 years, take-home naloxone (THN) programs have been implemented in various settings globally, and are considered an important public health intervention to reduce opioid overdose harm including mortality (1, 2). While THN programs have been found effective in reducing overdose deaths when optimized and implemented on a large-scale (3, 4), researchers argue that THN programs still have not reached their full potential (1, 5, 6). This has been attributed to among other things as not being sufficiently widely implemented throughout communities, too modest distribution compared to the clinical need and lack of adequate pre-supplied naloxone (1, 5, 6). Further, increasing overdose mortality rates underline the importance of continuing to scale up efficient THN implementation strategies (7–10).

Scaling-up evidence-based public health interventions may reduce behavioral health problems on a population level (11). While researchers and implementers have developed guides, models, and frameworks for scaling-up public health interventions (11–15), the subject is still one of the major questions in prevention science (11). Zomahoun et al. (16) describe six potential pitfalls associated with the scaling-up of evidence-based interventions, including cost-effectiveness, top-down implementation and contextual issues. Facilitators for success have been identified as strong leadership, broad engagement among implementers, and tailoring to local contexts (17). Specific THN implementation barriers are related to workflow, logistics, staff roles and responsibilities, education, engagement and trainings (18, 19). Among facilitators for success were leadership support, basic education and training efforts, and simple access to the actual naloxone kit for clients at no cost (18).

The emergence of digital tools in public health have provided new possibilities for infrastructure and outreach (20, 21). The use of digital technologies in health promotion provide inexpensive means to collect and assess health data on an individual level, and may influence behavioral change on a population level (20). By applying digital technology to reinforce health and health care, e-health has become a significant part of the healthcare system, providing more efficient services and accuracy (21, 22). Like e-health, “e-learning” (learning facilitated by the application of information technology, communication, and electronic media) has been applied to numerous settings within healthcare (23–25). E-learning is regarded as a cost-effective facilitator for both learning and knowledge integration into practice (23, 26, 27). In addition, e-learning is accessible to a large audience, and thus may be an appropriate tool for scaling-up public health interventions (20, 25, 28).Other potential benefits are related to potential of unlimited access to complete, and repeat courses, and the consistency and streamlining of trainings.

While e-learning is a promising invention with an almost unlimited reach, there is limited research on the use of e-learning while scaling up staff trainings for THN. Simmons et al. (29) found that an online opioid overdose prevention training was both feasible and acceptable among first responders in Pennsylvania, and a well-suited tool for rapid expansion. Lai Joyce Chun et al. (30) found that most community pharmacists in Australia preferred online trainings or webinars to face-to-face sessions. However, neither assessed pre-test pos*t*-test knowledge, or the training's impact on engagement and subsequent naloxone distribution. While widespread access and acceptability from stakeholders (such as staff members and health professionals) is important when scaling up, subsequent naloxone distribution is a main desirable outcome (31, 32). In-person train-the-trainer courses have shown to increase knowledge among stakeholders and engage in subsequent THN distribution (33–35). However, the use of e-learning in healthcare has come with concerns related to the quality of knowledge in different disciplines and settings, loss of traditional face-to-face interactions, and poor engagement (20, 23, 36).

In Norway, a government-funded THN program has gradually expanded to increase naloxone accessibility throughout the country. When preparing for scaling-up of the THN program for national availability, an e-learning course was developed to replace the previous in-person staff training course. The objective was to improve staff training availability, and ultimately to improve naloxone accessibility on a national level. The aims of this paper are (a) to describe participant characteristics for those that completed a THN e-learning course, (b) compare opioid overdose knowledge scores before and after e-learning course completion, and (c) explore subsequent THN distribution by those trained.

## 2 Materials and methods

### 2.1 Study design

This is a quasi-experimental, longitudinal study with pre-tests and post-tests of individuals who completed the Norwegian THN program's e-learning course. The recruitment period was from April 2021 to May 2022, with a 1-week and 1-month follow-up period for each participant.

### 2.2 Setting and e-learning course

In 2014, the Norwegian government funded a multifaceted overdose prevention strategy (37). One of the main measures in the strategy was the introduction of a THN program. At the time of initiation, the THN program was piloted in the two cities with the highest overdose mortality rates in Norway: Oslo and Bergen. The objective was to implement THN in existing low-threshold services for people who use drugs, primarily among those outside of formal treatment services. Central coordinators would approach relevant services, facilitating them for distribution by conducting trainings and provide them with naloxone kits. Naloxone kits were distributed without individual prescription and were free of charge for clients. A face-to-face train-the-trainer course was developed to facilitate for large-scale distribution, and any staff members who were trained could distribute (33). Central coordinators conducted the 90-min staff trainings and provided guidance and support for new distribution sites through the implementation stage (33).

The program had continuous geographical expansion from 2016, and new municipalities were prioritized and included based on their annual overdose mortality numbers. By 2021, 63 municipalities were included in the program. At the time of this study, over 100 distribution sites were included, and approximately 1,500 staff members had attended the face-to-face train-the-trainer course since program initiation (38). Distribution sites expanded to include additional low-threshold facilities, treatment centers, street outreach, and prisons. Throughout the expansion, the face-to-face train-the-trainer trainings became unsustainable to meet the growing program's needs. The program therefore introduced an e-learning course to improve staff training availability, and ultimately to improve naloxone accessibility on a national level.

The original in-person train-the-trainer course was adapted into a seven-module e-learning course. Each module covered different topics: (1) introduction, (2) the scope of the overdose problem, (3) opioid overdoses and naloxone, (4) overdose prevention, (5) overdose response, (6) summary and practical information, (7) project documentation. Quizzes were embedded throughout. When all modules were completed, a final exam became available. The exam was adapted from the opioid overdose knowledge scale (OOKS) and consisted of a multiple choice and checkbox questionnaire, examining course participants in opioid overdose knowledge. Completing the course and the exam would take an estimated 30–50 min. Completing the exam was a prerequisite for a staff member to distribute naloxone. Those who completed the exam received a course completion certificate.

### 2.3 Procedure

The e-learning course is available through the THN website for anyone to participate. When the e-learning course was launched, project coordinators recruited potential participants by informing both existing and future distribution sites via e-mail. Information on the new e-learning course was also posted on the THN program's Facebook page. All course participants had to register with an e-mail address and a mobile phone number to access the course.

During the study period, all course participants were directed to the study information sheet and consent form. Consenting participants were directed to the initial study questionnaire and the OOKS pre-test before they were directed to the e-learning course. Participants were not informed whether their answers were correct or incorrect. All modules were accessible for repetitions at any time. The integrated exam (accessible after all modules were completed) had to be passed (80% correct answers) for the post-test and follow-up questionnaires to be sent out. If a participant did not pass the exam, they could retake it as many times they wanted in order to pass.

A link with the OOKS post-test and 1-week follow-up questionnaire were sent out via email 1 week after the exam was passed. A link with the 1-month follow-up questionnaire was sent out 1 month after the exam was passed. Those who did not complete the 1-week follow-up would still receive the 1 month-follow-up questionnaire.

### 2.4 Participants

Participants in this study were individuals who completed the e-learning course (with the integrated exam) and completed pre- and post- tests. Participation was voluntary and anyone who accessed the e-learning course could complete the training.

### 2.5 Opioid overdose knowledge scale

The OOKS was used for the pre-test, the exam and the post-test. The OOKS assesses knowledge using the following items: risk factors for overdose, signs of an overdose, response to an overdose, and naloxone use. The questionnaire has proven to be internally reliable (39). The OOKS was translated into Norwegian by the first author in close cooperation with the other authors. Two questions were removed from the original questionnaire as they applied to injectable naloxone, whereas this project uses only intranasal naloxone. This adjustment removed six points off the original scale, and consequently resulted in scores between 0 and 39 [risks (0–9), signs (0–10), action (0–11), naloxone use (0–9), and knowledge total (0–39)].

### 2.6 Variables

The initial questionnaire included the following variables: age, gender, region (by municipality), profession (nurse, social worker, physician, psychologist, other), employment sector (municipality health service, specialist health service, non-profit organization, private, prison, police/security, other), previous THN training (no, yes), and if yes: time since previous training (never, more than 6 months, last 6 months).

The follow-up questionnaire explored naloxone distribution 1 week and 1 month following e-learning completion. Those who reported naloxone distribution were asked to provide the number of kits distributed. If they reported no distribution, they were asked “why” choosing from the following alternatives: workplace not distributing, clients not interested, I do not feel competent and other. Those who answered “other” were asked to elaborate in a free-text box.

The free-text responses were systematically coded and grouped into the following additional categories: “workplace in process of distributing”, “naloxone use for staff only”, “not relevant for our clients”, “not relevant for job position”, “other colleagues have distributed”.

### 2.7 Statistical analysis

Frequency analyses were performed for participants completing the initial questionnaire and for those completing the follow-up questionnaire at 1 week and 1 month. For the free text responses, thematic analyses were used. The responses were coded into categories and included in the results for the follow-up questionnaire. Wilcoxon signed-rank test was performed for comparison between pre-test and post-test OOKS for all four items and total. Effect size was calculated using Cohen criteria (small effect 0.1, medium effect 0.3, and large effect 0.5) (40). All analyses were completed in IBM SPSS Statistics 28.

Further, considering that a proportion of those consenting for the study were lost to follow-up throughout the stages of the follow-up period, we conducted chi-square tests of differences between those completing and non-completers’ demographical variables gender, work sector, previous training and profession. This was also the case for the follow-up-losses between 1 week and 1 month. No significantly differences were measured, therefore analysis were not presented in the manuscript.

### 2.8 Ethical approval

The Norwegian Centre for Research Data (project number: 614874) confirmed that the project processed personal data in accordance with data collection legislation.

## 3 Results

### 3.1 Participant characteristics

**Figure 1:**
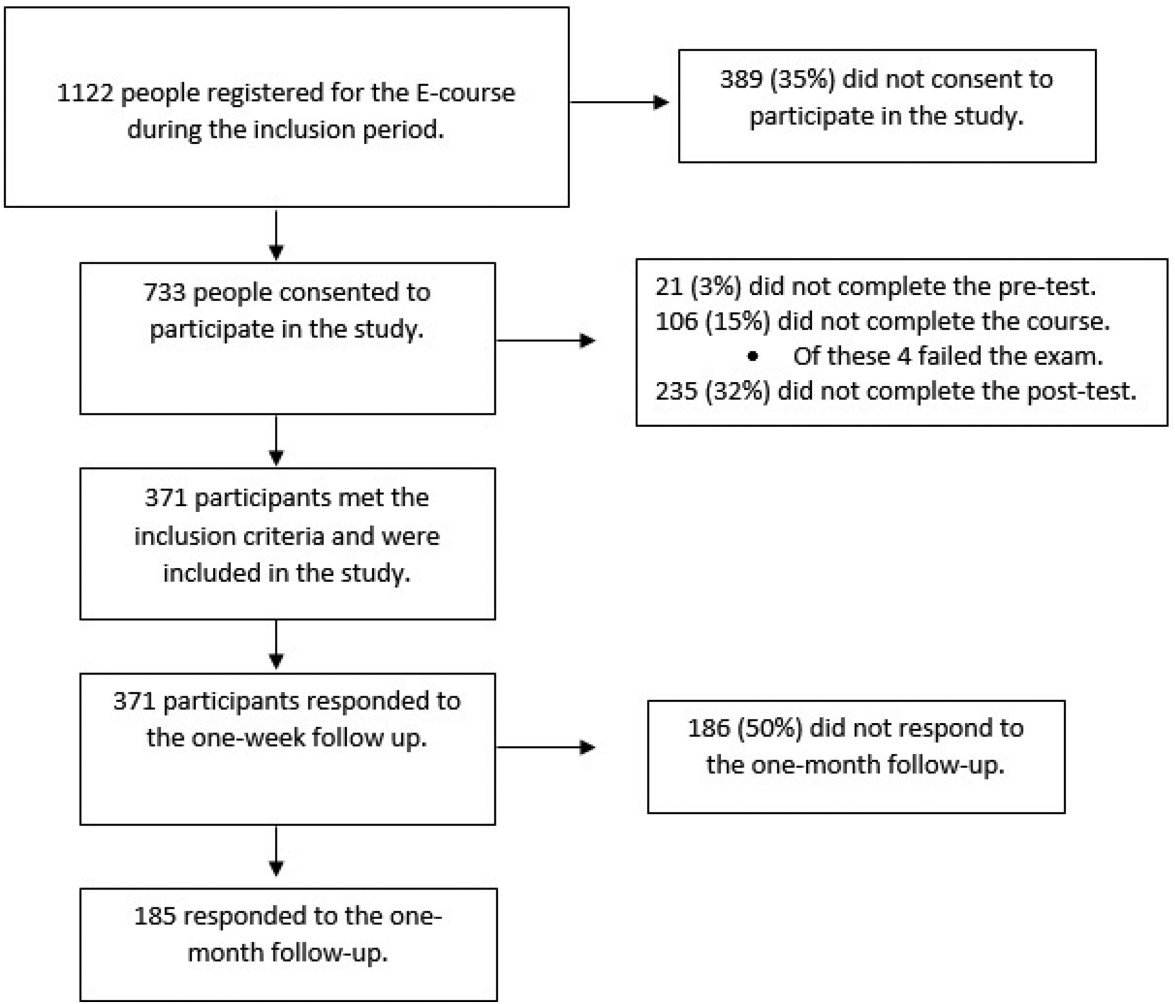
Flowchart of participants included in the study.

Of the 1,122 people registered for the e-learning course during the study period, 733 (65%) consented to participate in the study. Of the 733, 21 (3%) did not complete the pre-test, 106 (15%) did not complete the course (Four due to not passing the exam), and 235 (21%) did not complete the post-test. Thus, 371 (33%) persons completed the course, pre-test, and post-test and were included in the study (Figure 1).

The majority of the participants were female (*n* = 293, 79%) with a mean age of 41 years old (SD = 11.5) (Table 1). Nurses and social workers made up the two largest groups of professions (*n* = 277, 75%). Most of the participants were either employed within the municipality health service or the specialist health service (*n* = 310, 84%). Most had never previously attended a naloxone course (*n* = 321, 87%). Of those who previously attended a naloxone course, most reported that it was more than 6 months ago (*n* = 43, 86%).

**Table 1:** Participant characteristics (*n* = 371).

*N* (%)	
Gender	
Woman	293 (79.0)
Man	77 (20.8)
Missing	1 (0.3)
Age (SD)	41.3 (11.5)
Profession	
Nurse	147 (39.6)
Social worker	130 (35.0)
Physician	11 (3.0)
Psychologist	5 (1.3)
Other	77 (20.8)
Missing	1 (0.3)
Employment sector	
Municipality health service	195 (52.6)
Specialist health service	115 (31.0)
Private	19 (5.1)
Police/prison/security	19 (5.2)
Non-profit organizations	15 (4.0)
Other	7 (1.9)
Missing	1 (0.3)
Previous training	
No	321 (86.5)
Yes	49 (13.3)
Missing	1 (0.3)

SD, standard deviation.

### 3.2 Opioid overdose knowledge scores

There was a significant increase in knowledge for all items (Table 2). The items with the most improvement were “naloxone use” (*r* = −0.50) and “knowledge total” (*r* = −0.54), which both exhibited a large effect size. For the remaining items (risks, signs and action), the improved effect size was medium. The total average of correct answers increased from 85% in the pre-test to 94% in the post-test.

**Table 2:** Participant responses to opioid overdose knowledge scale, prior to and 1 week following e-learning course (*n* = 371).

Item (score)^a^	Pre-e-learning mean (SD)	Post-e-learning mean (SD)	Wilcoxon Z/*P*-value
Knowledge total (0–39)	33.06 (3.97)	36.81 (2.08)	−14.59, *P* < 0.001
Risks (out of 9)	7.44 (1.71)	8.45 (1.17)	−10.84, *P* < 0.001
Signs (out of 10)	8.09 (1.42)	8.70 (1.18)	−7.17, *P* < 0.001
Action (out of 11)	10.47 (0.77)	10.87 (0.39)	−8.81, *P* < 0.001
Naloxone use (out of 9)	7.06 (1.99)	8.78 (0.51)	−13.66, *P* < 0.001

Standard deviation (SD).

^a^ Two questions were removed from the original questionnaire from Williams et al. (39) as they applied to injectable naloxone.

### 3.3 Naloxone distribution 1 week and 1 month following course completion

There were 5% (*n* = 20) who reported naloxone distribution at 1-week follow-up, and 14.6% (*n* = 27) who reported naloxone distribution at 1-month follow-up (Table 3). Among those who reported no naloxone distribution at 1 week (*n* = 347, 94%), 5% (*n* = 19) reported to have distributed naloxone at 1 month. In addition, 60% (*n* = 12) of those who reported naloxone distribution at 1 week, did not respond to follow-up at 1 month. Consequently, though 27 individuals reported naloxone distribution at the 1-month follow-up, the total number of individuals reporting naloxone distribution during the follow-up period was 39 (10.5%). At 1-week follow-up, 20 participants reported to have distributed 50 naloxone kits. Further, at one-month follow-up an additional 44 kits had been distributed. Those who reported naloxone distribution at either time point reported a total of 94 naloxone kits distributed.

**Table 3:** Participant naloxone distribution and reasons for not distributing, at 1 week and 1-month follow-up.

	One week		One month	
	*N*	%	*N*	%
Did you distribute naloxone?				
Yes	20	5.4	27	14.6
No	347	93.5	158	85.4
Missing	4	1.1	0	0
Total	371	100	185	100
If no, why not?				
Clients not interested	137	39.5	62	39.2
Workplace not distributing	77	22.2	34	21.5
Workplace in process of distributing	58	16.7	20	12.7
Naloxone use for staff only	25	7.2	5	3.2
Not relevant for clients	14	4.0	9	5.7
Not relevant for job position	14	4.0	10	6.3
Other colleagues have distributed	5	1.4	3	1.9
I don't feel competent	4	1.2	2	1.3
Other	13	3.7	13	8.2
Total	347	100	158	100

Several reasons for not distributing were reported and listed in Table 3. After 1 week, 40% (*n* = 137) reported that “no clients had wanted naloxone”, and 39% reported either that “workplace not distributing” or “workplace in process of distributing”.

## 4 Discussion

We found that a THN staff e-learning course reached out to relevant stakeholders, and that participants increased opioid overdose knowledge scores for all items, particularly for items relating to naloxone use. The mean overall knowledge scores increased by 9% points from pre-test to post-test (from 85% to 94%). At 1-week follow-up 20 participants reported to have distributed 50 naloxone kits. Further, at 1-month follow-up an additional 44 kits had been distributed. Consequently, within the 1-month follow-up period, 39 participants reported to have distributed 94 naloxone kits.

In 13 months, the e-learning course reached out to three quarters of what the preceding face-to-face train-the-trainer-course did in 4 years (*n* = 1,122 vs. *n* = 1,500) (38). Most of the participants were either social workers or nurses (*n* = 277, 75%) within the health service, suggesting that the e-learning course reached out to relevant target groups (32, 41). In an earlier stage of the program, Madah-Amiri, Clausen and Lobmaier (33) found that 69% of those trained in the in-person train-the-trainer course were either nurses or social workers. While the wide-reach potential of e-learning is a key element when scaling-up, concerns related to the quality of knowledge transfer in different disciplines and settings have been raised (23). In this study, we found similar increase in participant pre- test, post-test knowledge as described by others when assessing similar trainings in classroom settings (33, 34). While Madah-Amiri, Clausen and Lobmaier (33) found a slightly larger effect size in the items “naloxone use” and “overall knowledge”, participants in our study exhibited higher mean knowledge level in both pre-test and post-test. The high pre-test knowledge scores may indicate an accumulation of opioid overdose knowledge among professional health workers due to 7 years of the ongoing national overdose prevention strategy. Unlike Madah-Amiri, Clausen and Lobmaier (33) and Dahlem et al. (34) who measured post-test scores immediately after course completion, our findings found a high degree of knowledge retained 1 week after course completion.

In addition to train relevant target groups, reaching sufficient naloxone coverage is important for naloxone programs to make an impact (3, 31, 42, 43). We found that 39 (10.4%) participants reported to have distributed 94 naloxone kits within the follow-up month, which totals 36% of the monthly distribution rates in the THN program during the same period^1^. At the 1-month follow-up 15% of respondents reported to have distributed naloxone, threefold the proportion reporting distribution after 1 week. These findings are similar to what others have found elsewhere assessing in-person settings; Orfaly et al. (44) found that 20% of those trained in a train-the-trainer program conducted trainings within a 6 months follow-up period. Further, in relation to number of kits distributed, Dahlem et al. (34) found that training 109 participants in their train-the-trainer scheme resulted in a total of 137 naloxone kit distributions within a 6 month follow-up period.

We found that the most frequent reasons for not distributing naloxone were “clients not interested”, “workplace not distributing” and “workplace in process of distributing”. The former suggests that enquires have been made, implying some level of site interest. However, the proportion of participants reporting “workplace not distributing” and “workplace in process of distributing” may indicate a need to better streamline the training and implementation processes. The importance of role clarity, tailoring into local contexts, and in-hand naloxone-accessibility have been described by others as facilitating factors for implementation, staff engagement and subsequent naloxone distribution (17, 18, 45). Taken into account our findings, we support the importance of robust preparation, local adjustments, and particularly in-hand naloxone availability at e-learning completion. Further, through the expansion of the program, the coordinator role changed with the introduction of the e-learning course. The loss of face-to-face interactions due to the shift from in-person trainings to e-learning, seem to have led to less robust hands on support and implementation planning. Thus, our findings echo the benefit of a central facilitator, as suggested by others, to provide both guidance and support at the implementation stage, and to add flexibility to the e-learning (23, 46). Additionally, to further improve the scaling-up process, it has been recommended that the use of digital tools should be accompanied by resources for supplies and staffing, monitoring of overdose events to predict potential naloxone demand and provide support for people with lived experiences to become naloxone trainers (47).

There are several limitations to this study. The recruitment strategy was a nonrandom convenience sample, with no inclusion criteria in terms of professional background, and the e-learning course was open for anyone to take, which may have affected, representativeness, test-scores and subsequent naloxone distribution rates. Further, considering that a notable proportion of those consenting were lost either before course completion, or during the follow-up period, the study may have been prone to a loss-to-follow-up bias. However, no demographical differences between completers and non-completers were found when conducting chi-square tests, thus loss-to-follow-up bias was not likely to distort the results in a major way. Secondly, the relatively large proportion of participants reporting either “workplace not distributing” or “workplace in process of distributing”, may suggest that a longer follow-up period may have captured a more complete picture on subsequent staff engagement. Further, the validated OOKS-questionnaire was subject to some changes; we translated the questionnaire into Norwegian and removed two questions not relevant to the nasal device used in the program. In terms of translation, there are no validated OOKS-questionnaire in Norwegian. but validity have been tested for other languages than English (48). Despite these limitations, the study also had several strengths. The study was able to reach out to a large number of relevant stakeholders and staff members. Our pre-test-post-test longitudinal cohort design allowed us to assess data at three different time points. While others have assessed train-the-trainer classroom settings, our study provide novel information on both knowledge transfer and subsequent naloxone distribution succeeding an e-learning course. Further, by assessing reasons for not distributing naloxone, the findings may be used to inform future implementation and practices.

## 5 Conclusions

This study found that an e-learning course reached out to relevant stakeholders such as nurses and social workers working at relevant services for PWUD. Additionally, the participants who completed the THN e-learning course increased their knowledge in all items, to near completely correct levels. Our findings suggest that an e-learning course is equally effective in terms of knowledge transfer as an in-person classroom setting, and may provide engagement in terms of naloxone distribution. Further, our findings support that the e-learning delivery model effectively contributed staff education for the scaling-up of a national THN program. However, our findings also emphasize the importance of clear implementation routines, including support from central coordinators to optimize the implementation process.

## Data Availability

The raw data supporting the conclusions of this article will be made available by the authors, without undue reservation.

## Footnotes

^1^Norwegian Take-Home Naloxone Program. Naloxone Distribution Data. Oslo: University of Oslo (2014–2022). (retrieved December 04, 2022).
